# Career Aspiration Fulfillment and COVID-19 Vaccination Intention among Nigerian Youth: An Instrumental Variable Approach

**DOI:** 10.3390/ijerph19169813

**Published:** 2022-08-09

**Authors:** Abayomi Samuel Oyekale

**Affiliations:** Department of Agricultural Economics and Extension, North-West University Mafikeng Campus, Mmabatho 2735, South Africa; asoyekale@gmail.com

**Keywords:** COVID-19, vaccination, career aspiration, dream jobs, Nigeria

## Abstract

COVID-19 is a pandemic of economic significance in the world. Vaccination has been identified as one of the veritable means to address the problem. Few studies have focused on youths’ vaccination intentions and the role of career aspiration fulfillment. This study therefore analyzed the effect engagement with dream jobs has on the willingness to get vaccinated for COVID-19. The data were from the 12th wave of the Nigeria COVID-19 National Longitudinal Phone Survey (NLPS) collected from 974 youths 15–25 years old. Instrumental Variable Probit regression was used for data analysis. The results showed that 86.57% and 80.34% of the rural and urban youths were willing to take the vaccines, respectively. Moreover, 31.18% and 25.94% of urban and rural youths, respectively, were engaged in their dream jobs. The Probit regression results showed that engagement with dream jobs was positively and significantly influenced (*p* < 0.01) by knowing someone who has a dream job, age and residence in the Southwest zone, while having a formal education reduced it. Instrumental Variable Probit regression results showed that willingness to take COVID-19 vaccine was positively influenced (*p* < 0.05) by having a dream job and intending to migrate to rural areas, while urban residence, age and residence in southern geopolitical zones decreased it. It was concluded that having a dream job promotes acceptance of COVID-19 vaccines among the youths, and there is a need for interventions to address hesitancy among urban youths and those in the southern geopolitical zones.

## 1. Introduction

Globally, COVID-19 remains a significant economic development hurdle. The pandemic has bred an array of health emergencies and economic crises that have distorted the modus operandi of several economic activities. There is a general consensus among policy makers that the pandemic is indisputably thwarting the attainment of several Sustainable Development Goals (SDGs). In some developing countries, this will also compromise anticipated long-term improvements in economic development indicators [1]. However, given the recent wane in the rate of infections and the availability of vaccines, policy makers are now hopeful of significant economic recovery even as nations adjust to living with the virus while the pandemic lasts. The onus therefore rests on policy makers to understand the post-COVID appropriate economic recovery pathways without further jeopardizing economic growth and human capital development through additional economic lockdowns.

In Nigeria, the post-COVID economic recovery policy framework seeks a multifaceted development model that is sufficiently resilient to covariate shocks and sustainable in the medium- and long-term period [2]. This is a fundamental prerequisite for a country that had not fully recovered from economic recessions when the COVID-19 pandemic struck. Specifically, between 2014 and 2016, the Nigerian economy witnessed a rapid decline in the average GDP growth rates, while the growth rate was −1.794% between 2019 and 2020 [3]. There are now indications of improvements in some Nigerian economic indicators, and the International Monetary Fund (IMF) recently projected a growth rate of 3.1% in 2023 [4]. Moreover, Nigerian policy makers cannot hold the prospects of sustainable economic recovery to mere political aspirations, given a recent World Bank publication that indicated that 95.1 million Nigerians will live below the poverty line in 2022 [5]. 

Globally, COVID-19 has led to significant job losses in different economic sectors. In Nigeria, job losses significantly impacted affected households due to the absence of efficient social protection programmes. Although the Nigerian government works to provide decent jobs for the growing youth population as a way to achieve some SDGs, very little progress had been made [6,7,8,9]. Specifically, the unemployment rate among youths—aged 15–34 years—increased from 7% in 2010 to 35% in 2020, while underemployment increased from 22% to 26% between 2010 and 2018, respectively [6]. The revised Nigerian Youths Employment Action Plan (NIYEAP) (2021–2024) seeks to address the impacts of COVID-19 on unemployment among Nigerian youths by contributing holistically to the government’s intention of creating 3.7 million jobs annually between 2019 and 2023, as constitutionally proposed in the 2019 National Youths Policy [6]. This is to enhance youths’ career aspirations, which are conceptually motivated by several factors including socio-demographic (gender [10,11], ethnicity [11], poverty status [12,13], educational achievements [9,14,15,16]), socio-psychological [14] and personality [17,18,19]. 

It should be noted that although COVID-19 disrupted the smooth operation of jobs across Nigeria, the government’s efforts at reducing its impact through the promotion of vaccination has met some stiff opposition [20]. In general, vaccination against COVID-19 promises a quick restoration of safety in the workplace and society at large through rapid development of herd immunity. However, misinformation on vaccine safety, political disinformation and some unfounded conspiracy theories remain the main hinderances to COVID-19 vaccine acceptability [20,21,22,23,24,25,26,27,28]. Due to very low response, moreover, the Nigerian government threatened to make the vaccines compulsory for all eligible citizens. A policy statement was issued to enforce vaccination for all government workers beginning from 1 December 2021 [29]. Although the government’s resolution to force workers to either get vaccinated or lose their jobs has been described as unconstitutional [30], the dilemma of becoming jobless due to vaccine hesitancy remains a paramount concern. 

Although some studies have been conducted on job satisfaction among Nigerian workers [7,8], the underlying determinants of career goal achievement among youths, particularly in the context of COVID-19 vaccination, are not well researched in the literature. Specifically, the decision to be vaccinated against COVID-19 can be motivated by several factors, among which engagement in dream jobs can be fundamental. In as much as some studies have analysed the effect of employment status on COVID-19 vaccine hesitancy [31,32,33,34,35], emerging literature on hesitancy towards COVID-19 vaccines has not fully considered the employment context of the problem [36]. While there have been mixed results on the role of employment status in COVID-19 vaccine hesitancy, it is still unclear how engagement in a dream job would impact the COVID-19 vaccination decision in the midst of legislative threats to mandate vaccination. This is a pertinent question in Nigeria, given the high unemployment rate and the fact that some employers are already mandating vaccination for their workers. This research therefore fills a major gap in the literature by analysing the effect of having a dream job—which is taken as the fulfillment of career aspirations—on COVID-19 vaccine hesitancy among Nigerian youths. The research is obviously pertinent to Nigeria’s current quest for post-COVID economic recovery, through human capital development and the provision of decent jobs. 

In some previous studies, employment status was found to influence hesitancy towards COVID-19 vaccination [32,33,34,35,36]. Specifically, Marzo et al. [35] found that COVID-19 vaccine hesitancy was significantly higher among employed people when compared with students. This is in alignment with the finding of Mayer et al. [36], Dror et al. [37], Khubchandani et al. [38], Stojanovic et al. [39] and Truong et al. [40] who found that employed people had lower probability of being vaccinated. It should be noted that one fundamental change after the lockdowns and states of disaster were lifted in many countries was inclusion of COVID-19 vaccination as one of the requirements for job seekers [41]. Among the general adult population, other correlates of vaccine hesitancy include gender, ethnicity, educational attainments, age and sources of information [42,43,44,45]. In some previous studies, Willis et al. [46] found that hesitancy towards COVID-19 vaccination among youths was not significantly influenced by gender, age, parental education, ethnicity and hours spent playing video games. 

Moreover, the role of career aspiration achievement in COVID-19 vaccine hesitancy is rarely studied. This is important because in some instances, the gradual return of normalcy in the work environment compels mandatory vaccination for the working class. Since good health is also fundamental to labour productivity, the role of vaccines in the context of disease prevention cannot be overemphasized. Similarly, understanding vaccine hesitancy among youths is essential because of their perceived less vulnerability to COVID-19 infection and high tendency to access misinformation on different social media. This is important because youths can also influence vaccination intentions and decisions in the adult population. This study therefore hypothesized that having a dream job does not significantly reduce hesitancy towards COVID-19 vaccination among Nigerian youths.

## 2. Materials and Methods

### 2.1. Data and Sampling Procedures

The data used in this study were from the 12th wave of the Nigeria COVID-19 National Longitudinal Phone Surveys (COVID-19 NLPS 2020–2021). These surveys were sponsored by the World Bank to monitor the impact of the COVID-19 pandemic on Nigerian households. The surveys were implemented in 12 waves by the National Bureau of Statistics (NBS) between April 2020 and April 2021. The sampling frame comprised 4976 nationally representative respondents of the 4th wave of the 2018/2019 GHS-Panel survey. Because of lockdowns and social distancing regulations during the COVID-19 pandemic, telephone interviews were conducted. The sampling frame comprised 4934 households that provided their own phone numbers or some reference phone numbers. Of these 4934 households, the estimated sample size to ensure national representation was 1800. Due to an expected response rate of about 60%, 3000 households were targeted. A balanced sampling approach was adopted to randomly select samples that would retain the characteristics of the entire frame across every covariate. The variances of the estimates were reduced due to the adopted balancing and calibration approaches. The representative nature of the data was ensured by balancing the selected samples across some socio-economic characteristics, including gender of household heads, per capita consumption expenditure, education, state, sector of residence and household size [47].

The data were collected in 12 waves. During the 1st wave, 3000 households were contacted, but only 1950 successfully completed the survey. These 1950 households eventually formed the sampling frame for subsequent surveys. In the 2nd wave, out of the 1852 households that were reached, 1820 successfully completed the survey. During the 3rd wave, efforts were made to reach 1925 of the households from wave 1 without including the 25 households that had refused in wave 2. However, only 1837 households were reached, of which 1790 successfully completed the survey. The sample sizes for the other waves were 1691 in wave 4, 1656 in wave 5, 1640 in wave 6, 1573 in wave 7, 1547 in wave 8, 1533 in wave 9, 1497 in wave 10 and 1680 in wave 11, The 12th wave sought to interview youths aged 15–25 years within the households. Therefore, 1238 households were included in the survey, having met the requisite criterion for inclusion. In each of these households, a youth between 15 and 25 years of age was interviewed. In all, 995 youths were contacted, and the 974 that were captured in the database formed the units of data analyses in this study [47]. Sample weights were also generated for each of the respondents.

The survey was carried out by the National Bureau of Statistics (NBS) interviewers who had sufficient experience in telephone surveys and were familiar with the Computer Assisted Phone Interview (CAPI) using the Survey Solutions platform. Two-day trainings were organized for the interviewers to facilitate their understanding of the contents of the questionnaire. The trainings also incorporated some mock interviews, and the design of the questionnaire ensured that the survey did not last beyond 20 min. The questionnaire, which was originally designed in English was also translated into three major Nigerian languages (Yoruba, Ibo and Hausa). The interviewers had tablets with the correct date and time that had to be fully charged. Internet connections were provided to ensure access to the server of the NBS using their previously assigned usernames and passwords. The procedures for conducting the survey were application synchronization, assignment receipt, and conduct of the interview by dialing the phone numbers of selected households and completion of the survey [47].

### 2.2. Specification of Instrumental Variable Probit Model

Instrumental Variable Probit Regression was used for data analysis. The model’s specification begins with a specification of standard Probit regression model (Equation (1)):

(1) $$Prob\left(Y_{i}=1/X\right)=\int_{-\infty }^{X_{i}\beta }{\left(2\pi \right)}^{-1/2}\exp(-\frac{t^{2}}{2})dt=\phi \left(X_{i}\beta \right)$$

In the above equation, the cumulative distribution function of a standard normal variable is denoted as ϕ, the vector of *X* denotes the explanatory variables, and *β* is a vector of the estimated parameters. Equation (2) presents a reduced form of Equation (1):

(2) $$Y_{i}=\alpha +\overset{k}{\underset{i}{\sum }}\beta_{i}X_{i}+\gamma DJ_{i}+e_{i}$$

In Equation (2), $Y_{i}$ is the willingness to be vaccinated against COVID-19 (yes = 1, 0 otherwise). $X_{i}\;$ are the explanatory variables. DJ_i_ is the endogenous variable which is the engagement in dream jobs dummy variable (yes = 1, 0 otherwise). For this variable, the youths were asked a question that required a yes/no answer on whether they consider themselves to presently have the job of their dreams. In addition, $\;\alpha ,\;\beta_{i}\;$ and γ are the estimated parameters, while $e_{i}$ is the error term. If *Cov* ($e_{i},DJ$_*i*_) = 0, endogeneity is absence. If an endogeneity problem [*Cov* ($e_{i},DJ$_*i*_) ≠ 0] is present, the instrumental variable must be engaged in the specification of the vaccination model [48,49,50,51]. The dream job model is stated as:

(3) $$DJ_{i}=\delta +\overset{k}{\underset{i}{\sum }}\partial_{i}X_{i}+\mu KS_{i}+v_{i}$$

The variable, $KS_{i}$, in Equation (3) is knowing someone who fulfilled his or her career aspiration, which is the instrumental variable for estimating Equation (2). This variable was coded as yes = 1 and 0 otherwise. It should be noted that selection of instrument(s) is a major problem in the conduct of endogenous regression models. A major criterion is that selected instrument(s) must be correlated with the endogenous variable (DJ_*i*_) but uncorrelated with the dependent variable ($Y_{i}$). The vector of *X* explanatory variables comprises preference for migrating to capital cities (yes = 1, 0 otherwise), preference for migrating to other cities (yes = 1, 0 otherwise), preference for migrating to rural areas (yes = 1, 0 otherwise), preference for migrating to other countries (yes = 1, 0 otherwise), preference for migrating with no specific place (yes = 1, 0 otherwise), North East zone (yes = 1, 0 otherwise), North West zone (yes = 1, 0 otherwise), South East zone (yes = 1, 0 otherwise), South South zone (yes = 1, 0 otherwise), South West zone (yes = 1, 0 otherwise), urban resident (yes = 1, 0 otherwise), gender (male = 1, 0 otherwise), primary education (yes = 1, 0 otherwise), secondary education (yes = 1, 0 otherwise), tertiary education (yes = 1, 0 otherwise) and age of youths (years). The models were estimated with STATA 17 software. The Wald test of exogeneity was computed to determine if the career fulfillment variable was truly endogenous. If this statistic is statistically significant (*p* < 0.05), there is endogeneity, and estimation Equation (1) using the standard Probit regression model will produce inconsistent parameters.

## 3. Results 

### 3.1. Youths Demographic Characteristics, Dream Job Engagement and Vacine Hesitancy

Table 1 shows the descriptive characteristics youths’ selected demographic characteristics. The results show that 48.87% were males and 36.55% were residents in urban areas. Average age was 19.38 years. Formal education was not possessed by 10.78%, and 14.48% attained primary education. Secondary education was attained by 66.63%, while 8.11% had tertiary education. Across the geopolitical zones, respondents from the North East accounted for the highest proportion (22.90%) of the youths, while those from the South West followed with 18.99%. The geopolitical zones with the least respondents were South South and South East with 9.55% and 13.24%, respectively. In addition, although only 27.72% indicated having dream jobs, 71.77% knew someone who was doing his/her dream jobs.

**Figure 1:**
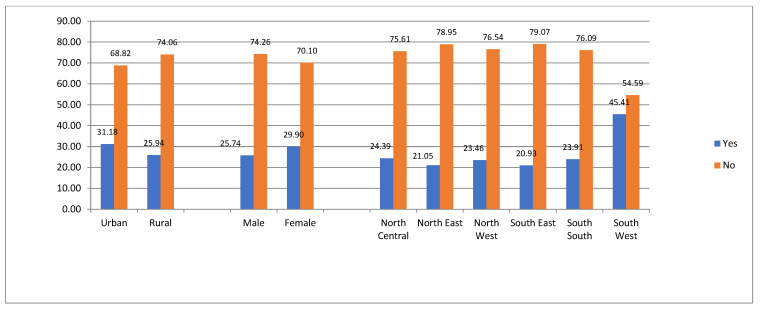
Distribution of engagement with dream jobs across youths’ selected demographic characteristics.

Figure 1 shows the respondents’ perceptions of engagement in dream jobs across some selected demographic characteristics. The figure shows that 31.18% and 25.94% of the urban and rural households indicated being engaged in their dream jobs, respectively. In addition, female respondents had higher fulfillment of their career aspirations with 29.90% against 25.74% for males. Across the geopolitical zones, respondents from the South West had the highest engagement with dream jobs (45.91%). This is followed by North West and North Central with 24.49% and 24.39%, respectively. However, youths from the South East had the least engagement with dream jobs (20.93%).

**Figure 2:**
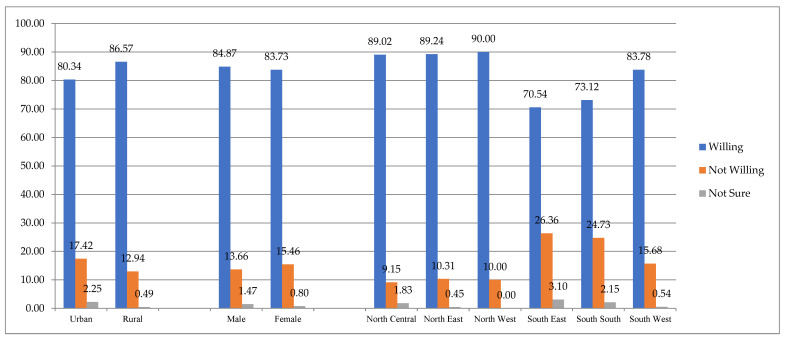
Youths’ willingness to take COVID-19 vaccines across selected demographic characteristics.

Figure 2 also shows the distribution of the youths’ selected demographic characteristics in relation to their willingness to take COVID-19 vaccines. It reveals that 80.34% and 86.57% of the youths from urban and rural areas were willing to take the vaccines. Specifically, 84.29% of all the youths were willing to take the vaccines. The gender dimension of their responses shows that 84.87% of the males and 83.73% of the females were willing to take the jabs. Across the geopolitical zones, youths from northern parts of the country were generally more willing to take the vaccines. Specifically, while 90% of the youths from the North West would take the vaccines, only 70.54% and 73.12% answered “yes” in the South East and South West zones, respectively.

### 3.2. Probit Regression Results of the Determinants of Dream Jobs Engagement

Table 2 shows the results of the Probit regression model for the determinants of engagement with dream jobs. The table shows the results for the standard probit regression parameters and the marginal parameters. Six of the included explanatory variables were statistically significant (*p* < 0.01), while one showed statistical significance at a 10% level. However, the model properly fitted the data since the likelihood ratio chi-square statistic showed statistical significance (*p* < 0.01). This indicates that the parameters of the included variables cannot be said to be jointly equal to zero. The results showed that the parameter of knowing someone who is engaged in their dream job showed statistical significance (*p* < 0.01) with a positive sign. The marginal parameter implies that knowing someone who is engaged with their dream job increased the expected probability of being engaged with a dream job by 0.1644, if other variables are taken as constant. Moreover, the estimated parameter for youths’ age is statistically significant (*p* < 0.01) with a positive sign. This implies that as age increased, the probability of having a dream job increased. The marginal parameter reveals that holding other variables constant, an increase of one year in the youths’ age will increase the expected probability of being engaged in dream jobs by 0.0185. The marginal parameter of preference to migrate to rural areas reveals that taking other variables as constant, the youths who had a preference to migrate to rural areas had their expected probability of being engaged in dream jobs significantly decline by 0.0632 (*p* < 0.10).

Furthermore, the parameters of all the education variables are statistically significant (*p* < 0.01) with a negative sign. These results indicate that attainment of formal education significantly reduced the probability of having a dream job. The marginal parameter for primary education reveals that taking other variables as constant, the youths with a primary education had their expected probability of being engaged in dream jobs reduced by 0.1360, when compared with those without any formal education. The marginal parameter for secondary education also reveals that taking other variables as constant, the youths with a secondary education had their expected probability of being engaged in dream jobs reduced by 0.1861 when compared with those without any formal education. Lastly, the marginal parameter for tertiary education reveals that taking other variables as constant, the youths with a tertiary education had their expected probability of being engaged in dream jobs reduced by 0.1559, when compared with those without any formal education.

Among the geopolitical zone variables, only the parameter for the South West zone showed statistical significance (*p* < 0.01). This indicates that compared to their counterparts from the North Central zone, youths from the South West zone had a higher probability of being engaged with dream jobs. Specifically, taking other variables as constant, the youths from the South West zone had their expected probability of being engaged in dream jobs higher by 0.2390 when compared with those from the North Central zone.

### 3.3. IV Probit Results of the Determinants of Youths Willingness to Take COVID-19 Vaccines

Table 3 shows the results of the instrumental variable Probit regression analysis. It reveals that the model produced a good fit of the data given the statistical significance of the Wald Chi Square statistics (*p* < 0.01). The computed parameter of the Wald test of exogeneity is statistically significant (*p* < 0.01). This implies that the engagement in dream job variable is truly endogenous and estimating a standard Probit regression would produce estimators that will be inconsistent. It should also be noted that the correlation coefficient (rho) between the error terms is −0.5982 and the athrho parameter is −0.6904 which is statistically significant (*p* < 0.05). All these confirm the endogeneity of the engagement of the dream job variable. 

The estimated parameter for the engagement with the dream job variable is statistically significant (*p* < 0.01) and with a positive sign. This shows that the probability of being willing to take the COVID-19 vaccine increases with engagement in dream jobs. The parameter of migrating preference to rural areas is also with a positive sign and statistically significant (*p* < 0.05). This implies that youths that would like to migrate to rural areas had a higher probability of being willing to take COVID-19 vaccines. The parameter of the South East geopolitical zone reveals that compared to their counterparts from the North-Central zone, the youths from the South East zone had a significantly lower probability of being willing to take COVID-19 vaccines (*p* < 0.01). Similarly, the parameter of the South South geopolitical zone reveals that compared to their counterparts from the North Central zone, the youths from the South South zone had significantly lower probability of being willing to take COVID-19 vaccines (*p* < 0.01). Moreover, compared to their counterparts from the North Central zone, the youths from the South West zone had a significantly lower probability of being willing to take COVID-19 vaccines (*p* < 0.05). 

The results also showed that as age increased, the probability of being willing to take COVID-19 vaccines decreased significantly (*p* < 0.01). Urban residents also had a significantly lower probability of willingness to take the vaccines (*p* < 0.10).

## 4. Discussion

### 4.1. Fulfillment of Career Aspirations among the Youths 

The results showed that engagement with dream jobs was reported by 27.72% of the youths. This percentage is quite low because some of the respondents were between the ages of 15 and 25 years and may be full-time students or apprentices. Although education and other trainings that often engage youths are pursued as long-term efforts targeted at achieving a particular outcome based on their career aspirations [52], educational attainments of the youths were low. Specifically, a majority held secondary school certificates, while only 8.11% had some form of tertiary education. The result may point to the underlying mismatch between the jobs that some youths are qualified for in the labour markets due to their low educational levels and their ultimate career goals. Given that aspirations sometimes stem from inherent skills or career trainings designed to maximize lifetime economic gains [53], others may have career goals that are unattainable due to limitations in their educational attainments. The low engagement with dream jobs among youths may also reflect rigours that are associated with climbing the ladder of career goal fulfillment in some occupations. 

Engagement in dream jobs among youths was enhanced by knowing someone who had fulfilled their career goal. This result emphasizes the vital role of role models and mentors in the ultimate fulfillment of youths’ career aspirations. It should be noted that several factors influence youths choices for role models. Sometimes, parameters such as international visibility, social influences, career success and wealth may inform the choice for youths with mindsets for career growth and excellence [54]. Such positive emulation facilitates the impact of mentorship among youths to promote their sound career aspirations through goal setting and constructive team spirit [55].

The results further showed that formal education reduced the likelihood having a dream job. This finding clearly underscores a perplexing mismatch between the career aspirations of some youths and their engagements in the labour markets due to the gross absence of decent jobs. The case in Nigeria is perplexing because labour unions are utilizing the power of industrial strikes and collective bargaining to increase the number of years they can work for the government without considering the future of the young generation. The outcome is a growing number of unemployed graduates. It should also be emphasized that illiterate youths may have career aspirations that would be tailored towards apprenticeship or vocational trainings, thereby making them relatively easy to achieve. The choice of career by some youths is influenced by a desire to be successful in life. This has often propelled schooling and relevant trainings [56]. However, the reality in the Nigerian labour market is perplexingly disappointing due to high unemployment and the inability of many qualified graduates to secure a much desired “white collar” job. 

In addition, the lack of significant industrial and business development in some Nigerian geopolitical zones aggravates the job scarcity saga. This has been worsened by several constraints such as unreliable electricity, dilapidated roads and national insecurity. Specifically, the results revealed that respondents from the South West zone had a higher likelihood of being engaged in dream jobs. Although unemployment prevails across the whole country, the concentration of many industries in many states in the South West zone, especially Lagos state, may have accounted for the engagement of some youths in their dream jobs. The result may have also underpinned the role of cultural values and diversity in career aspirations and achievements [57], given that some Nigerian ethnic groups are very industrious, while indolence characterizes some others. 

The results also showed the positive role that age plays in the achievement of career aspirations. This is noteworthy due to the complexity associated with decisions on individuals’ careers as age increases [57]. It is expected that the fulfillment of career aspirations will increase as people grow old because time is often required to attain the requisite education and qualifications. Similarly, it also takes some time to climb the ladder of professional cadres at which time some youths would begin to realize career fulfilment.

### 4.2. Youths’s Willingness to Take COVID-19 Vaccines

Vaccine hesitancy among youths is now an important research effort given the gradual extension of COVID-19 vaccines to the younger segments of the population [46]. The results showed that 84.29% of the youths were willing to get vaccinated. This is appreciably higher than the 42% that was reported by Willis et al. [42]. In another study, Rehati et al. [58] found that 60% of Chinese adolescents were willing to get vaccinated before the vaccines were available. In addition, Euser et al. [59] found that 73% of Dutch youths were willing to be vaccinated against COVID-19. The high acceptance of COVID-19 vaccines among the youths reflects the growing confidence in the safety of the vaccines given that a lot of people had already taken the jabs with no or very minimal side effects. 

The results further showed that engagement in a dream job increased the probability of willingness to get vaccinated. This could reflect the desirability of health investment and disease prevention behaviour by those with dream jobs. It is important to also note that the result may reflect workplace policies on COVID-19 vaccination that make vaccination compulsory for all workers. There have been different opinions on these directives from some employers. Although Hassan et al. [60] found that making COVID-19 vaccines compulsory for all citizens will reduce its acceptability among Nigerians, given a very high rate of unemployment in the country, workers may have no other option than taking the jab if it is made mandatory.

The result also showed that as age increased, willingness to take the vaccines decreased. This finding is contrary to the results of Willis et al. [46] who found age to be statistically insignificant in explaining COVID-19 vaccine hesitancy among youths. Moreover, Euser et al. [59] found willingness to be vaccinated against COVID-19 to significantly increase with age in Dutch youths. 

In addition, youths from urban areas had a significantly lower probability of being willing to be vaccinated, while those with an intention to migrate to rural areas had a higher probability. Rural dwellers in Nigeria have benefitted from several previous child vaccination programmes, and their access to some misinformation and conspiracy theories on COVID-19 and its vaccines may be limited. However, among the general populations, some previous authors have reported higher hesitancy towards COVID-19 vaccines among urban residents [61]. 

The results showed that youths from all the southern zones of Nigeria had a lower probability of accepting COVID-19 vaccines. 

## 5. Conclusions

This paper analysed the effect of engagement with dream jobs on COVID-19 vaccine hesitancy among Nigerian youths. The empirical analyses in this paper are important because of the recent extension of COVID-19 vaccines to youths and children in many countries. The study is particularly novel given its focus on an employment-related factor that was estimated endogenously as a correlate of COVID-19 vaccine hesitancy. The findings have emphasized the role of engagement with dream jobs in promoting acceptability of COVID-19 vaccines. Efforts to promote COVID-19 vaccination among youths should therefore be directed at the reorientation of urban youths and those from the southern parts of the country to recognize vaccine safety by dispelling prevailing misinformation and disinformation. In addition, the findings have highlighted the impact of role models and the focus on the creation of decent jobs that match educational attainments of youths.

The study, however, suffers from some limitations. The first is that the data were collected through phone interviews, which may have negatively impacted the quality since there were very few avenues to verify provided information. In addition, the data also suffer from non-responses that always characterize panel surveys as respondents become disinterested over time. Moreover, future studies can recursively analyse the correlates of youths’ vaccination status and engagement in dream jobs.

## Figures and Tables

**Table 1:** Descriptive statistics of selected youths’ demographic characteristics.

	Mean	Std. Err.	95% Conf. Interval	
			Lowest Bound	Uppermost Bound
Willing to be vaccinated (Yes = 1; 0 otherwise)	0.8429	-	-	-
Urban resident (Yes = 1, 0 otherwise)	0.3655	-	-	-
Gender (Male = 1, 0 otherwise)	0.4887	-	-	-
Age of youths (years)	19.3768	0.1014	19.1778	19.5758
No education (Yes = 1, 0 otherwise)	0.1078	-	-	-
Primary education (Yes = 1, 0 otherwise)	0.1448	-	-	-
Secondary education (Yes = 1, 0 otherwise)	0.6663	-	-	-
Tertiary education (Yes = 1, 0 otherwise)	0.0811	-	-	-
North Central zone (Yes = 1, 0 otherwise)	0.1684	-	-	-
North East zone (Yes = 1, 0 otherwise)	0.2290	-	-	-
North West zone (Yes = 1, 0 otherwise)	0.1848	-	-	-
South East zone (Yes = 1, 0 otherwise)	0.1324	-	-	-
South South zone (Yes = 1, 0 otherwise)	0.0955	-	-	-
South West zone (Yes = 1, 0 otherwise)	0.1899	-	-	-
Engaged with dream jobs (Yes = 1, 0 otherwise)	0.2772	-	-	-
Know someone with dream job (Yes = 1, 0 otherwise)	0.7177	-	-	-

**Table 2:** Probit regression results of the determinants of engagement in dream jobs.

	Standard Probit Parameters		Marginal Parameters	
Variables	Coefficients	*z*-Statistics	dy/dx	*z*-Statistics
Know someone with dream job (Yes = 1, 0 otherwise)	0.5616 ***	5.11	0.1644 ***	5.75
Migrating to capital cities (Yes = 1, 0 otherwise)	0.1175	1.06	0.0376	1.06
Migrating to other cities (Yes = 1, 0 otherwise)	0.0089	0.08	0.0029	0.08
Migrating to rural areas (Yes = 1, 0 otherwise)	−0.2020	−1.62	−0.0632 *	−1.66
Migrating to other countries (Yes = 1, 0 otherwise)	−0.0752	−0.68	−0.0241	−0.68
Migrating to no specific place (Yes = 1, 0 otherwise)	−0.0464	−0.28	−0.0147	−0.28
Urban resident (Yes =1, 0 otherwise)	0.0160	0.15	0.0051	0.15
Gender (Male = 1, 0 otherwise)	0.0532	0.57	0.0171	0.57
Age of youths (years)	0.0576 ***	5.41	0.0185 ***	5.43
Primary education (Yes = 1, 0 otherwise)	−0.4821 ***	−2.67	−0.1360 ***	−3.12
Secondary education (Yes = 1, 0 otherwise)	−0.5524 ***	−3.57	−0.1861 ***	−3.46
Tertiary education (Yes = 1, 0 otherwise)	−0.5902 ***	−2.76	−0.1559 ***	−3.53
North East zone (Yes = 1, 0 otherwise)	−0.0587	−0.39	−0.0187	−0.39
North West zone (Yes = 1, 0 otherwise)	−0.0832	−0.51	−0.0262	−0.52
South East zone (Yes =1, 0 otherwise)	−0.0142	−0.08	−0.0045	−0.08
South South zone (Yes = 1, 0 otherwise)	0.1389	0.74	0.0462	0.71
South West zone (Yes = 1, 0 otherwise)	0.6728 ***	4.25	0.2390 ***	4.02
Constant	−1.9650 ***	−4.87		
Diagnostic statistics				
Number of observations	974			
LR chi2(17)	115.73 ***			
Log likelihood	−517.08			

***—statistically significant at 1%, *—significant at 10%.

**Table 3:** Instrumental variable probit results for the determinants of willingness to take COVID-19 vaccines.

	Coefficient	Std Error	*z*-Statistics
Engagement with dream job (Yes = 1, 0 otherwise)	1.233 ***	0.4349	2.84
Migrating with capital cities preference (Yes = 1, 0 otherwise)	−0.0025	0.1140	−0.02
Migrating with other cities preference (Yes = 1, 0 otherwise)	0.0777	0.1115	0.70
Migrating with rural areas preference (Yes = 1, 0 otherwise)	0.2780 **	0.1283	2.17
Migrating to countries preference (Yes = 1, 0 otherwise)	0.0756	0.1118	0.68
Migrating no specific place preference (Yes = 1, 0 otherwise)	0.2543	0.1691	1.50
North East zone (Yes = 1, 0 otherwise)	−0.0682	0.1597	−0.43
North West zone (Yes = 1, 0 otherwise)	−0.0138	0.1707	−0.08
South East zone (Yes = 1, 0 otherwise)	−0.6166 ***	0.1889	−3.26
South South zone (Yes = 1, 0 otherwise)	−0.5573 ***	0.1883	−2.96
South West zone (Yes = 1, 0 otherwise)	−0.3825 **	0.1849	−2.07
Urban resident (Yes = 1, 0 otherwise)	−0.2021 *	0.1077	−1.88
Gender (Male = 1, 0 otherwise)	−0.0217	0.0932	−0.23
Primary education (Yes = 1, 0 otherwise)	0.0659	0.2122	0.31
Secondary education (Yes = 1, 0 otherwise)	0.1239	0.1927	0.64
Tertiary education (Yes = 1, 0 otherwise)	−0.1140	0.2659	−0.43
Age of youths (years)	−0.0290 **	0.0140	−2.07
Constant	0.7948 *	0.4142	1.92
Athrho	−0.6904 ***	0.2691	−2.57
Lnsigma	−0.8650 ***	0.0227	−38.18
Rho	−0.5982 ***	0.1728	
Sigma	0.4210	0.0095	
Other diagnostic statistics			
Number of observations	974		
Wald chi2(17)	94.41 ***		
Wald test of exogeneity	6.58 ***		

***—significant at 1%; **—significant at 5%; *—significant at 10%.

## Data Availability

The data used for this study are in public domain. The data were downloaded from https://microdata.worldbank.org/index.php/catalog/3712/study-description#metadata-data_access (accessed on 4 March 2022).
